# Eslicarbazepine, but Not Lamotrigine or Ranolazine, Shows Anticonvulsant Efficacy in Carbamazepine-Resistant Rats Developed by Window-Pentylenetetrazole Kindling

**DOI:** 10.3390/brainsci12050629

**Published:** 2022-05-11

**Authors:** Cecilia Zavala-Tecuapetla, Joaquín Manjarrez-Marmolejo, Josué Orlando Ramírez-Jarquín, Claudia Verónica Rivera-Cerecedo

**Affiliations:** 1 Laboratory of Physiology of Reticular Formation, National Institute of Neurology and Neurosurgery, Insurgentes Sur 3877, La Fama, Mexico City 14269, Mexico; manjarrezmj@yahoo.com.mx; 2 Department of Molecular Neuropathology, Institute of Cellular Physiology, National Autonomous University of Mexico, Circuito Exterior s/n, Ciudad Universitaria, Mexico City 04510, Mexico; jjarquin@ifc.unam.mx; 3 Bioterium Academic Unit, Institute of Cellular Physiology, National Autonomous University of Mexico, Circuito Exterior s/n, Ciudad Universitaria, Mexico City 04510, Mexico; crivera@ifc.unam.mx

**Keywords:** Drug-Resistant Epilepsy, pentylenetetrazole, carbamazepine, anticonvulsant effect, lamotrigine, eslicarbazepine, ranolazine

## Abstract

Approximately 30% of epileptic patients develop Drug-Resistant Epilepsy. Based on evidence that shows a loss of efficacy in some sodium channel blocker antiseizure drugs in epilepsy, we focus our study on assessing the anticonvulsant efficacy of different sodium channel blockers on carbamazepine (CBZ)-resistant seizures generated using the window-pentylenetetrazole (PTZ) kindling model to verify whether one of these drugs presents some anticonvulsant effect that could have potential therapeutic use. Wistar rats were treated with a subthreshold dose of PTZ (35 mg/kg) three times/week. Fully kindled rats were then treated with a single dose of CBZ (40 mg/kg i.p.) at 2, 9 and 16 days after their last kindling stimulation to obtain CBZ-resistant rats. Right after, sodium channel blockers were tested for anticonvulsant action (lamotrigine, 30 mg/kg i.p.; eslicarbazepine, 150 or 300 mg/kg i.p.; ranolazine, 10, 20 or 40 mg/kg i.p.). Behavioral parameters included severity, latency or duration of convulsions. Our data showed for the first time directly that eslicarbazepine does have an anticonvulsant effect over CBZ-resistant seizures, while lamotrigine shows drug resistance and ranolazine demonstrates severe seizure worsening. It is of potential therapeutic relevance that eslicarbazepine could be useful to control seizures resistant to common sodium channel blockers such as CBZ.

## 1. Introduction

Epilepsy is a recurrent and progressive neurological disease, affecting approximately 1–2% of the global population [1]. Antiseizure drugs (ASDs) therapy is initially employed as a treatment for epilepsy. However, approximately 30% of epileptic patients do not respond effectively to ASDs and consequently develop Drug-Resistant Epilepsy [2,3]. Therefore, there is a great necessity to study new therapeutic targets and develop new ASDs against Drug-Resistant Epilepsy.

It is of clinical relevance that the efficacy of sodium channel blocker ASDs, such as carbamazepine (CBZ), phenytoin and lamotrigine, appear to be impaired in epilepsy, which has been observed in both humans and rodent models [4,5,6,7]. The above is probably due to reduced sensitivity of the sodium channels to the ASDs as a result of changes in the properties of these channels, which may contribute to the development of drug resistance [8]. These data prompted us to evaluate the potential anticonvulsant effects of sodium channel blocker ASDs in CBZ-resistant rats [9].

To investigate Drug-Resistant Epilepsy at the preclinical level, there are tools such as chronic animal models (e.g., lamotrigine-resistant kindled animals, phenytoin-selected kindled animals, etc.) [10] which have contributed to the study of both the pathological mechanisms involved and the research of new drugs with therapeutic potential.

Recently, we reported the use of the window-pentylenetetrazole (PTZ) kindling as a model to generate CBZ-resistant rats [9], which is developed by the application of a smaller number of PTZ administrations necessary to reach the kindled state [11] and by a single exposure to sodium channel blockers such as CBZ, immediately after (post-kindling phase), to generate drug-resistant seizures [12], features that make this model technically simple and minimally invasive, facilitating the generation of drug-resistant animals. In this sense, this animal model can be suitable as an alternate preclinical approach to predict the effectivity of potential drugs against drug-resistant seizures.

Whereas traditional sodium channel blocker ASDs such as CBZ, phenytoin and lamotrigine block the opening of sodium channels by acting on its fast inactivation state and thus avoiding further generation of action potentials in neurons [13], the effects of eslicarbazepine, a new sodium channel blocker, are on the slow inactivation state of these channels, therefore limiting sustained repetitive neuronal firing [13,14]. Other drugs such as ranolazine (an antianginal drug) preferentially blocks the persistent sodium current suppressing the repetitive firing of neurons [15,16]. Both drugs, eslicarbazepine and ranolazine, have showed potential anticonvulsant activity [15,16,17,18,19,20,21,22,23,24,25,26].

Based on the above, here we aimed to find out whether lamotrigine, eslicarbazepine or ranolazine (all sodium channel blockers), have or not anticonvulsant effectiveness specifically on CBZ-resistant rats developed by the window-PTZ kindling model.

## 2. Materials and Methods

### 2.1. Animals

The study was performed in male Wistar rats, weighing 250–300 g. The rats were maintained under standard laboratory conditions: 12 h light/dark cycles; temperature, 22 °C; with free access to standard diet and water. The minimum number of necessary animals were used, avoiding unnecessary suffering to them. All animal procedures were performed with the approval of the Internal Committee for the Care and Use of Laboratory Animals of the National Institute of Neurology and Neurosurgery M.V.S. (project 25/16) and in accordance with the Mexican Official Norm (NOM-062-ZOO-1999). All experiments complied with the National Institutes of Health Guide for the Care and Use of Laboratory Animals (NIH Publications No. 80-23, revised 1996).

### 2.2. Chemicals

Pentylenetetrazole (PTZ) and ranolazine (ranolazine dihydrochloride) were dissolved in 0.9% saline solution. Eslicarbazepine [(S)-(-)-10-acetoxy-10,11-dihydro-5H-dibenz/b,f/azepine-5-carboxamide; BIA 2-093], lamotrigine and carbamazepine (CBZ) were each dissolved in 0.3% dimethyl sulfoxide (DMSO, *v*/*v*). All drugs were purchased from Sigma (Sigma-Aldrich, St. Louis, MO, USA).

### 2.3. Development of the Window-PTZ Kindling Model

We used the window-PTZ kindling model previously reported [9,11]. With this model a lower number of PTZ injections is necessary to develop fully kindled rats, and then to develop drug-resistant seizures [9,11]. Briefly, rats were administered with a subthreshold dose of PTZ (35 mg/kg i.p., volume of 1 mL/kg body weight) three times/week [27]. The PTZ administration scheme was as follows: rats received 4 initial administrations; during the next 10 assays rats did not receive PTZ administrations, and the treatment finished with 3 final PTZ administrations. After each PTZ administration, the convulsive behavior was evaluated for 20 min. Using the Racine modified scale [28,29], the kindling development was assessed: 0: no response; 1: ear and facial twitching, sniffing, blinking; 2: nodding or myoclonic body jerks; 3: clonus of one forelimb, bilateral forelimb clonus; 4: rearing with bilateral forelimb clonus; 5: generalized clonic-tonic convulsions with loss of postural control. When rats achieved the criterion of three consecutive stage 4 or 5 seizures, they were considered fully kindled animals. If the rats were not successfully kindled within 17 sessions (no-kindled rats), they were excluded from our study.

### 2.4. Generation of CBZ Resistance in Kindled Rats

As it was previously reported [9,12], to determine CBZ resistance in the fully kindled rats (*n* = 48), they were treated with a single dose of CBZ (40 mg/kg i.p.) at 2, 9 and 16 days after their last kindling stimulation. CBZ was administered 60 min prior to stimulation with PTZ (35 mg/kg). The convulsive behavior was evaluated for 20 min after each PTZ administration [9,12].

### 2.5. Evaluation of Sodium Channel Blockers in CBZ-Resistant Rats

To assess whether sodium channel blockers could or could not have anticonvulsant effects on CBZ-resistant rats, a second drug trial was performed one week after the last administration of CBZ (day 23):

- Lamotrigine group (*n* = 6), rats received lamotrigine (30 mg/kg i.p.) 70 min prior to stimulation with PTZ (35 mg/kg i.p.);
- Eslicarbazepine group (*n* = 12), here we tested two doses of eslicarbazepine (150 or 300 mg/kg i.p.) each of which were administered 40 min prior to PTZ stimulation;
- Ranolazine group (*n* = 30), in this group we tested three doses of ranolazine (10, 20 or 40 mg/kg i.p.) that were administered 30 min prior to PTZ stimulation.

Behavioral evaluation included severity of convulsions, and latency to stage 2 or 4/5 convulsions. Duration was only measured when a stage 4 or 5 convulsion occurred. Previous studies reported that these doses of sodium channel blockers were able to reduce the severity of convulsions [12,20,21,26].

### 2.6. Statistical Analysis

For statistical analysis and graph preparation, we employed GraphPad Prism 5.01 (GraphPad software, La Jolla, CA, USA). Data are presented as the mean ± standard error mean (S.E.M.). Significance was considered as a *p* value of <0.05. The results of kindling development or between different administrations of CBZ in the convulsion severity, were analyzed using a one-way ANOVA with repeated measures and Tukey’s post hoc test for multiple comparisons. CBZ-resistant state (16 d) and drug treatment with each sodium channel blocker (23 d) were compared by Student’s *t*-test for paired replicates.

## 3. Results

### 3.1. Generation of Carbamazepine-Resistant Fully Kindled Rats

**Figure 1:**
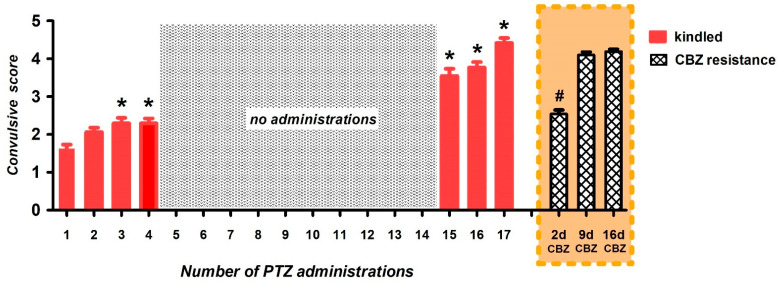
To induce window-PTZ kindling, rats were administered with a subthreshold dose of PTZ (35 mg/kg i.p.) three times/week. With this protocol, we obtained fully kindled rats (red bars). Next, fully kindled rats were treated with a single dose of CBZ (40 mg/kg i.p.) at 2, 9 and 16 days after their last kindling stimulation (orange box), thus obtaining CBZ-resistant rats (grid bars). Data represent mean ± SEM; * *p* < 0.05, which is significantly different from the first assay in the kindled group; # *p* < 0.05, which is significantly different from the kindled condition. Pentylenetetrazole (PTZ), carbamazepine (CBZ).

Throughout the induction of the window-PTZ kindling, the rats showed an increased susceptibility to convulsions as noted by the progression of convulsive score from stage 1–3 to stage 4–5 convulsions (Figure 1). From a total of 84 animals, 48 rats (57%) were kindled, 23 rats (27%) were no-kindled and 13 rats (16%) died during kindling development.

The kindled rats presented stage 1–3 convulsions (convulsive score 2.3 ± 0.13; Figure 1) during the first four administrations of PTZ, and achieved a stage 4 or 5 convulsions (convulsive score 4.5 ± 0.11; Figure 1) in response to the last three PTZ administrations, settling into full kindled state.

Subsequently, the administration of CBZ (40 mg/kg) reduced the convulsive score two days (2 d) after the last kindling stimulation (from 4.5 ± 0.11 to 2.5 ± 0.11; *p* < 0.001; Figure 1) in response to PTZ stimulation, that it was observed in 100% of animals tested (48/48). However, at day 9, a second dose of CBZ failed to reduce the convulsive score, that is, the rats now presented resistance to the same dose of CBZ (convulsive score 4.1 ± 0.07; Figure 1). With a third administration of CBZ at day 16, we did not observe a change in the convulsive score, with persisting stage 4/5 convulsions (convulsive score 4.2 ± 0.06; Figure 1) in 100% of animals tested. In this way, we obtained CBZ-resistant rats.

### 3.2. Effect of Sodium Channel Blockers on Convulsions Generated by PTZ in CBZ-Resistant Rats

To evaluate whether treatment with sodium channel blockers may acutely suppress convulsions in CBZ-resistant rats, these animals were treated with one of the following drugs: lamotrigine (30 mg/kg i.p.), eslicarbazepine (150 or 300 mg/kg i.p.) or ranolazine (10, 20 or 40 mg/kg i.p.).

**Figure 2:**
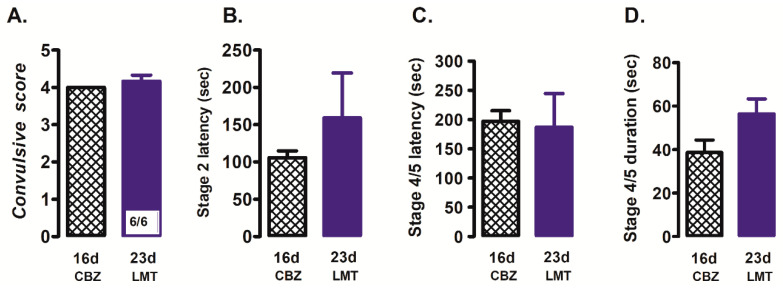
Effects of lamotrigine (23 d; LMT, 30 mg/kg i.p.) on convulsive score (**A**), stage 2 latency (**B**), latency (**C**) and duration (**D**) of the stage 4/5 convulsions with respect to the CBZ-resistant state (16 d). Data represent mean ± SEM. Carbamazepine (CBZ).

With lamotrigine, CBZ-resistant rats (6/6, 100% of rats; Figure 2A) developed stage 4/5 convulsions in response to PTZ stimulation (23 d; Figure 2A). Lamotrigine did not manage to modify significantly the convulsive score (23 d; 4.2 ± 0.17) with respect to the CBZ-resistant state (16 d; 4.0 ± 0.0) (Figure 2A), nor any other parameter evaluated (latency or duration of convulsions; Figure 2B–D).

**Figure 3:**
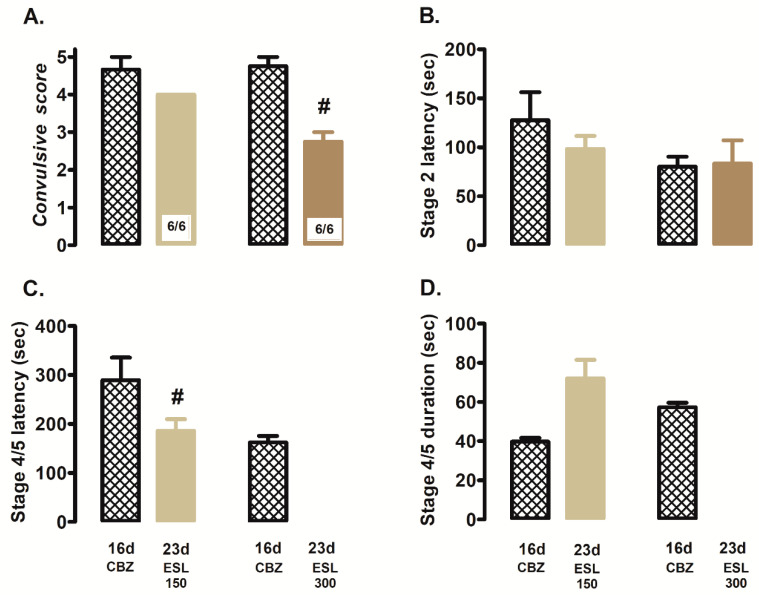
Effects of eslicarbazepine (23 d; ESL, 150 or 300 mg/kg i.p.) on convulsive score (**A**), stage 2 latency (**B**), latency (**C**) and duration (**D**) of stage 4/5 convulsions. Data represent mean ± SEM. # *p* < 0.05, significantly different from the CBZ-resistant state (16 d). Carbamazepine (CBZ).

Similar results were obtained with the administration of eslicarbazepine at a dose of 150 mg/kg, which did not avoid the stage 4/5 convulsions in the 100% of CBZ-resistant rats (23d, 6/6; Figure 3A). There was only a significant decrease in the latency of stage 4/5 convulsions (23 d; 186.3 ± 23.6 s) with respect to the CBZ-resistant state (16 d; 289.3 ± 46.1 s; *p* = 0.04; Figure 3C). Neither stage 2 convulsions latency (Figure 3B) nor the duration of stage 4/5 convulsions (Figure 3D) were significantly modified. However, with the dose of 300 mg/kg, eslicarbazepine significantly prevented the generalized seizures, decreasing the convulsive score in 100% of animals (6/6) in response to PTZ stimulation (23 d; 2.75 ± 0.25) (Figure 3A,C,D) with respect to the CBZ-resistant state (16 d; 4.8 ± 0.30; *p* = 0.001; Figure 3A). There was not change at stage 2 convulsions latency in these animals (Figure 3B).

**Figure 4:**
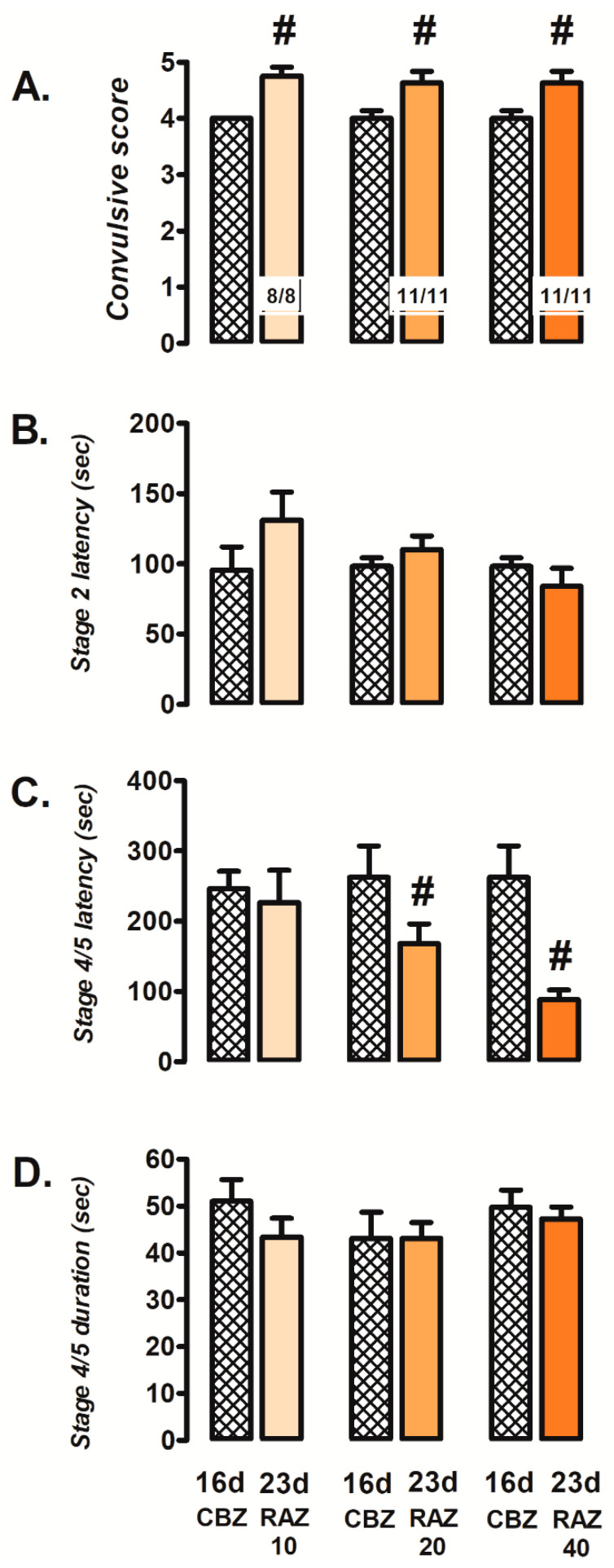
Effects of ranolazine (23 d; RAZ, 10, 20 or 40 mg/kg i.p.) on convulsive score (**A**), stage 2 latency (**B**), latency (**C**) and duration (**D**) of stage 4/5 convulsions. Data represent mean ± SEM. # *p* < 0.05, significantly different from the CBZ-resistant state (16 d). Carbamazepine (CBZ).

As for the effect of ranolazine, the three doses tested (10, 20 or 40 mg/kg) demonstrated severe seizure worsening, promoting the stage 4/5 convulsions in CBZ-resistant rats (Figure 4A). With the dose of 10 mg/kg (8/8), ranolazine increased the convulsive score (23 d; 4.75 ± 0.16) with respect to the CBZ-resistant state (16 d; 4.0 ± 0.01; *p* = 0.003; Figure 4A). Neither stage 2 convulsions latency (Figure 4B) nor the latency or duration of stage 4/5 convulsions (Figure 4C,D) were modified at this dose. However, with the dose of 20 mg/kg (11/11), ranolazine not only increased the convulsive score (23 d; 4.64 ± 0.20) with respect to the CBZ-resistant state (16 d; 4.0 ± 0.14, *p* = 0.03; Figure 4A), but it also managed to reduce the latency of stage 4/5 convulsions (23 d; 168.2 ± 28.1 s) with respect to the CBZ-resistant state (16 d; 262.4 ± 44.7 s; *p* = 0.03; Figure 4C). No changes were observed in the remaining parameters (Figure 4B,D). Finally, similar results were observed with the dose of 40 mg/kg (11/11), where ranolazine increased the convulsive score (23 d; 4.63 ± 0.23) with respect to the CBZ-resistant state (16 d; 4.0 ± 0.13, *p* = 0.04; Figure 4A), and also achieved a significant decrease latency of stage 4/5 convulsions (23 d; 88.3 ± 13.9 s) with respect to the CBZ-resistant state (16 d; 262.4 ± 44.6 s; *p* = 0.02; Figure 4C). Neither stage 2 convulsions latency (Figure 4B) nor the duration of stage 4/5 convulsions (Figure 4D) were modified at this dose.

Thus, with the exception of eslicarbazepine-300, sodium channel blockers had no anticonvulsant efficacy on CBZ-resistant animals.

## 4. Discussion

In this study, we investigated whether or not some sodium channel blockers could have a potential therapeutic impact for treating Drug-Resistant Epilepsy.

Specifically, our results showed that in CBZ-resistant animals generated by the window-PTZ kindling, they also exhibit drug resistance to the sodium channel blocker lamotrigine, while with ranolazine, they presented proconvulsive effects, and with the higher dose of eslicarbazepine tested, they showed anticonvulsive effects.

CBZ-resistant animals showed a subsequent drug resistance to lamotrigine (23 d) after its acute administration. Lamotrigine is a conventional sodium channel blocker that acts by acting on its fast inactivation state in the same way that CBZ does [13], and by inhibiting the release of excitatory amino acids such as glutamate [30]. Our findings are in line with previous reports with lamotrigine-resistant kindled animals [7,31,32] that suggest that drug resistance might be extended to other drugs with a similar mechanism of action (in our study, sodium channel blockers) [9,12,31].

In the case of eslicarbazepine, this drug showed an acute anticonvulsant efficacy at the higher dose employed (300 mg/kg). The application of eslicarbazepine blocked the generalized seizures by managing to control the spread of convulsive activity on CBZ-resistant animals. Our data confirm previous evidence that eslicarbazepine could have anticonvulsant properties in epilepsy and Drug-Resistant Epilepsy [18,19,20,21,23,24].

Eslicarbazepine is a third-generation antiseizure drug approved as adjunctive treatment for focal seizures with or without secondary generalization [18]. This drug is a sodium channel blocker with effects that differ from those described for conventional ASDs such as CBZ or lamotrigine. Eslicarbazepine acts on the slow inactivation state of sodium channels, hence limiting sustained repetitive neuronal firing [14,33,34].

An explanation for the drug resistance observed with CBZ and lamotrigine and the anticonvulsant effect observed with eslicarbazepine in this model could be the different way in which these drugs affect the sodium channel as we previously mentioned (differential effects on the fast and slow inactivation state of the channel). Another explanation for the different effects observed between these drugs could be an alteration of the sodium channels subunits, a proposed mechanism in the development of Drug-Resistant Epilepsy [8,35,36,37]. A loss of CBZ efficacy on sodium channels lacking β subunits has been observed [38], while eslicarbazepine does not seem to alter its effectiveness [34]. However, further research concerning changes on the sodium channel inactivation state or changes in the sodium channel subunit conformation, and its correlate with its altered functional properties, are necessary to provide additional information with respect to the mechanisms of drug resistance in CBZ-resistant animals, before any mechanistic conclusions can be drawn.

Therefore, application of eslicarbazepine could be a particularly efficacious therapy in drug-resistant epileptic patients that no longer respond to CBZ’s anticonvulsant effect [17,18,21,22].

Moreover, even though it has been reported that the eslicarbazepine dose tested, which was 150 mg/kg, produces behavioral impairments on lamotrigine-resistant kindled rats [31], we do not observe any behavioral changes on CBZ-resistant rats, with an eslicarbazepine dose of 300 mg/kg. We do not rule out that these differences depend on the different strain of rats (Sprague-Dawley/Wistar).

Additionally, it should be taken into account that eslicarbazepine also inhibits the high- and low-affinity hCaV3.2 inward currents and the T-type CaV3.2 channels [20,39], and therefore these effects could influence the anticonvulsive effect observed here in the CBZ-resistant animals.

With respect to ranolazine, it had a clear proconvulsive effect by promoting the fast generalization of clonic-tonic seizures in CBZ-resistant animals, reducing in a dose-dependent manner the time it takes for seizures to develop, but without being fatal. This is in contrast to studies suggesting that preferential inhibition of the persistent component of the sodium current in neuronal sodium channels exerts an anticonvulsant effect [25,26,40,41,42,43].

However, why does ranolazine seem to worsen seizures in CBZ-resistant rats? Ranolazine, an approved drug that displays antiarrhythmic properties [44,45], has been demonstrated to block the persistent sodium current evoked by mutations of sodium channels [15,25,26]. In this way, ranolazine could exert an anticonvulsant effect through its action on these channel mutations, while a completely different effect over the channel subunit type and conformation, may aggravate seizures and epilepsy condition, just as we observed a proconvulsant effect caused by ranolazine on CBZ-resistant animals. Whether the persistent sodium current is increased or not in CBZ-resistant animals, and how this potential increase in persistent sodium current is probably due to a particular sodium channel mutation in this model, are questions that require further studies.

Additionally, why are seizures not fatal in CBZ-resistant rats? It was notorious that no drug-resistant animals died as a consequence of generalized seizures after treatment with ranolazine. This data could be an indirect effect of ranolazine on the heart because of its antiarrhythmic properties [44,45], and probably protects against sudden death as a result of generalized seizures [46,47,48]. It is worth mentioning that sudden unexpected death is one of the main causes of mortality among patients with Drug-Resistant Epilepsy [46,49], where ranolazine could have an interesting effect, but more studies are needed to verify this idea.

Finally, it has been reported that lamotrigine and eslicarbazepine have a reduced efflux transport from the brain by P-glycoprotein at clinically relevant concentrations, consequently affecting its therapeutic effect [50,51]. We have reported previously that CBZ resistance could be associated with alterations in the protein levels of efflux transporters such as P-glycoprotein at the blood–brain barrier [9]. This previous data could be associated with the lamotrige resistance observed in CBZ-resistant animals impacting its effectiveness. However, the anticonvulsive effect of eslicarbazepine was not affected in CBZ-resistant animals, so we do not rule out that other mechanisms are also involved in this phenomenon of drug resistance, which requires further investigation [8,35].

## 5. Conclusions

Our data showed for the first time directly that eslicarbazepine does have an anticonvulsant effect over CBZ-resistant seizures, while lamotrigine shows that drug resistance and ranolazine demonstrate severe seizure worsening.

As we mentioned previously, Drug-Resistant Epilepsy is a multifactorial phenomenon, where not only alterations in brain drug uptake, but also alterations in brain drug targets, such as alterations in the sodium channel subunits, may be participating in its development. This last mechanism remains to be determined in future studies in this model to establish its possible association with the generation of Drug-Resistant Epilepsy.

A limitation of our current study is that we evaluated the effects of sodium channel blockers by acute administration. Therefore, it is necessary to know whether its differential effects described herein would continue or change with a chronic administration.

Understanding the reasons why seizures become resistant to sodium channel blockers may therefore provide insights into new therapeutic targets with the end goal of finding new potential drugs to treat drug-resistant epileptic patients that do not respond to conventional sodium channel blockers such as CBZ and in whom eslicarbazepine could be an effective adjunctive therapy.

## Data Availability

Data are available on request from the corresponding author.

## Abbreviations

PTZ	pentylenetetrazole
CBZ	carbamazepine
